# Phenotypic characterization of SETD3 knockout Drosophila

**DOI:** 10.1371/journal.pone.0201609

**Published:** 2018-08-01

**Authors:** Marcel Tiebe, Marilena Lutz, Dan Levy, Aurelio A. Teleman

**Affiliations:** 1 German Cancer Research Center (DKFZ), Heidelberg, Germany; 2 Heidelberg University, Heidelberg, Germany; 3 The Shraga Segal Department of Microbiology, Immunology and Genetics, Ben-Gurion University of the Negev, Be’er-Sheva, Israel; 4 National Institute for Biotechnology in the Negev, Ben-Gurion University of the Negev, Be’er-Sheva, Israel; Biocenter, Universität Würzburg, GERMANY

## Abstract

Lysine methylation is a reversible post-translational modification that affects protein function. Lysine methylation is involved in regulating the function of both histone and non-histone proteins, thereby influencing both cellular transcription and the activation of signaling pathways. To date, only a few lysine methyltransferases have been studied in depth. Here, we study the *Drosophila* homolog of the human lysine methyltransferase SETD3, CG32732/dSETD3. Since mammalian SETD3 is involved in cell proliferation, we tested the effect of dSETD3 on proliferation and growth of *Drosophila* S2 cells and whole flies. Knockdown of dSETD3 did not alter mTORC1 activity nor proliferation rate of S2 cells. Complete knock-out of dSETD3 in *Drosophila* flies did not affect their weight, growth rate or fertility. dSETD3 KO flies showed normal responses to starvation and hypoxia. In sum, we could not identify any clear phenotypes for SETD3 knockout animals, indicating that additional work will be required to elucidate the molecular and physiological function of this highly conserved enzyme.

## Introduction

Lysine methylation is a reversible protein modification that can be considered to have a signaling function, similar to protein phosphorylation [1–3]. Lysine residues can accept up to three methyl groups, thereby forming mono-, di-, and tri-methylated derivatives (me1, me2 and me3). Each state of methylation creates a unique signature that can act to recruit specific trans-acting factors, thus triggering downstream signaling events. Traditionally, protein lysine methylation has been studied in the context of histone biology. Lysine methylation of histones plays a key role in regulating DNA compaction and transcription, and alterations in histone lysine methylation are involved in several pathologies including cancer [4–8]. Lysine methylation, however, also plays a role outside the context of histones (“non-histone methylation”). Indeed, several non-histone proteins have been shown to be methylated by lysine methyltransferases [9–15]. Lysine methyltransferases can be divided into two groups, the SET domain containing proteins and the 7β-strand enzymes. The majority of lysine methylations are catalyzed by SET-domain proteins [9, 16, 17]. The SET domain name was derived from three *Drosophila* genes (Su(var)3-9, enhancer of zeste, trithorax) which all have lysine methyltransferase activity and share this domain [18–22]. 55 human proteins contain a SET domain, however many of them are not studied in depth via animal loss-of-function experiments [23]. One such lysine methyltransferase is SETD3.

SETD3 is a methyltransferase that can methylate histones, specifically at H3K36 and H3K4. Indeed some functions of SETD3 appear to be related to transcriptional regulation: SETD3 was first described in zebrafish where overexpression of SETD3 leads to decreased cell viability and induction of apoptosis [24]. Subsequently in mouse C2C12 myoblasts SETD3 was found to play a role in muscle development [25]. SETD3 levels increase during muscle differentiation in C2C12 cells *in vitro*, and SETD3 is required for their differentiation [25]. Indeed, overexpression of SETD3 is sufficient to induce muscle differentiation [25]. SETD3 was shown, generally, to induce transcription and to physically interact with the myogenic transcription factor MyoD to induce transcription of myogenin [24, 25]. SETD3 is also regulated by the cell cycle. Protein levels of SETD3 are highest in S-phase and lowest in M-phase in HeLa cells [26]. SETD3 knockdown causes reduced proliferation and reduced soft-agar colony formation of HepG2 cells, and conversely, overexpression of SETD3 increases their proliferation rate and soft-agar colony formation [26]. In addition to its histone-related functions, SETD3 has been shown to methylate non-histone targets, such as FoxM1 [27]. In this context SETD3 was shown to suppress the expression of VEGF upon hypoxia in HeLa cells and SETD3 protein levels were also suppressed by hypoxia [27].

Interestingly, SETD3 has been linked to cancer in various ways. SETD3 is thought to be involved in the carcinogenesis of B-cell lymphomas [28]. A truncated and catalytically inactive form of SETD3 was found to be expressed in one case of CXP lymphoma, a Xrcc4 and p53 deficient mouse B-lineage lymphoma model. When overexpressed, this truncated version of SETD3 is able to induce colony growth of NIH3T3 cells in soft agar assays [28, 29]. A further indication that SETD3 might play a role in cancer is the finding that SETD3 is overexpressed at the mRNA and protein level in Renal cell tumors (RCTs) [30]. Intriguingly, despite the overexpression of SETD3 in RCTs, SETD3 expression levels correlate positively with survival, suggesting SETD3’s role in carcinogenesis might be complex [30]. Similar to RCTs, SETD3 protein levels were also found to be elevated in hepatocellular carcinoma (HCC) and even positively correlate with increasing stages of liver cancers [26].

Despite this work, however, no SETD3 loss-of-function animals have been described.

Thus, it is currently not known whether SETD3 is required for cell survival or organ growth *in vivo*. Similarly, even though SETD3 is involved in the response to hypoxia in various cancer cell lines, it is not known whether SETD3 is required for an appropriate hypoxia response in normal cells or the whole organism. Given that SETD3 can methylate histone and non-histone targets, it is also not known what impact a lack of SETD3 has on global mRNA levels, and whether the main function of SETD3 is related to histone methylation or non-histone methylation. In sum, to understand the physiological role of SETD3, it is important to study SETD3 knockout animals.

Since *Drosophila* is an excellent model for uncovering gene function, due to the high level of functional conservation to humans and to the powerful genetic toolbox that is available in the fly [31], we decided to study *Drosophila* lacking SETD3.

## Results

### Bioinformatic identification of Drosophila SETD3

To identify the *Drosophila* homolog of human SETD3 we blasted the protein sequence of human SETD3 (NP_115609) against the *Drosophila* proteome and the uncharacterized SET-domain protein CG32732 was the highest hit with a sequence similarity of 41.4% (S1 Fig) [32, 33]. Conversely, blasting the protein sequence of CG32732 against the human RefSeq protein database resulted in hSETD3 being the highest scoring hit [34]. Thus, we conclude there is a clear orthology between human SETD3 and *Drosophila* CG32732, and we refer to CG32732 as the *Drosophila* homolog of SETD3, dSETD3. To look further at the evolutionary relationship between CG32732 and human SET-domain containing proteins, we aligned several human SET-domain members [35] together with CG32732 using Clustal Omega [36] and then submitted the alignment to phylogenetic tree analysis [37]. As expected CG32732 clusters most tightly with human SETD3 (S1B Fig).

### SETD3 knockdown with two different RNAi lines in the wing causes undergrowth

**Fig 1:**
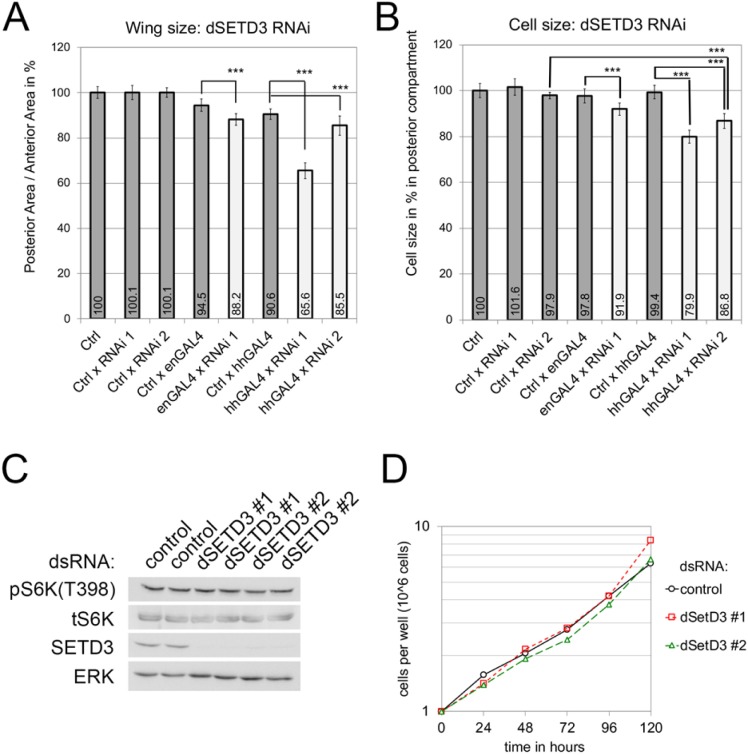
Knockdown of dSETD3 in the wing disc reduces growth. **(A-B)** Knockdown of dSETD3 in the posterior compartment of the wing decreases tissue size. (A) Size of the posterior compartment normalized to size of the control anterior compartment. (B) Cell size in the posterior compartment was determined by counting the number of cells via their trichomes in a defined area, and then calculating the inverse–ie area per cell. Animals were raised at 25°C, 10 wings per genotype were used for quantifications. Error bars: standard deviation (SD); *** *t* test < 0.001 **(C)** Knockdown of dSETD3 in Drosophila S2 cells does not change mTORC1 activity. dSETD3 was knocked-down by treating S2 cells with 2 independent dsRNAs for 5 days, and mTORC1 activity was assayed via immunoblotting for phosphorylation of the direct target S6K. The two lanes per sample represent biological duplicates. **(D)** S2 cells treated with 3 different dsRNAs against dSETD3 do not show a change in cell proliferation rates. S2 cells were treated with dsRNAs for 5 days and plated freshly at the same cell density. Cell number was then counted every 24 hours for 5 days.

Since SETD3 is overexpressed in Renal cell tumors [30] and SETD3 protein levels correlate positively with the proliferation rate of liver cancer cells [26], we first asked whether SETD3 regulates cell growth or proliferation *in vivo* in the animal. To this end, we tested the effect of dSETD3 knockdown in the *Drosophila* wing. The fly wing has been used extensively to identify genes regulating tissue growth, because wing size can be quantified very sensitively due to the wing’s large size and flat morphology. We used two different inducible RNAi lines to knockdown dSETD3 expression in the posterior compartment of the wing during development, using either hedgehog-Gal4 (hhGAL4) or engrailed-Gal4 (enGAL4) (see S2 Fig for expression domain). We then quantified the area of the posterior compartment of the wing, relative to the anterior compartment which serves as a normalization control. Knockdown of dSETD3 using RNAi line 1 caused a significant reduction in tissue size (Fig 1A). The reduction in tissue size was stronger with hhGAL4 than with enGAL4 (Fig 1A), in line with the fact hhGAL4 is a stronger GAL4 driver and therefore drives expression of the inducible RNAi more strongly than enGAL4. Expression of RNAi line 2 in the posterior compartment also caused a significant reduction in tissue size (Fig 1A). Since the two RNAi lines are independent and non-overlapping, this is usually taken as an indication that an off-target effect is unlikely.

Smaller tissue size can result from a smaller number of cells, or from reduced cell size, or from a combination of both. Thus, we also measured the effect of dSETD3 knockdown on cell size by quantifying cell density by counting trichomes in a defined region of the posterior adult wing. SETD3 knockdown caused a significant reduction in cell size with both RNAi lines (Fig 1B). The reduction in cell size was similar in magnitude to the reduction in overall tissue size (Fig 1A), with the exception of hhGAL4 x RNAi 1 where the reduction in tissue size was greater than the cell size reduction. In sum, these data suggest that dSETD3 knockdown in the wing causes reduced tissue size, mainly due to an effect on cell size with some contribution from reduced cell proliferation. As described below, however, we surprisingly did not find a size effect in dSETD3 knockout animals, raising the question how these RNAi data should be interpreted (see Discussion below).

### SETD3 Knockdown in Drosophila S2 cells

*Drosophila* S2 cells are another system often used to study both cell size and activity of mTORC1, the main kinase responsible for regulating cell size [38]. We knocked-down expression of dSETD3 in S2 cells using two independent dsRNAs, which knockdown SETD3 expression quite efficiently (Fig 1C). Knockdown of dSETD3 did not cause a decrease in mTORC1 activity, assayed using phosphorylation of a direct, canonical mTORC1 target, S6K (Fig 1C). Knockdown of dSETD3 also did not affect cell proliferation rates (Fig 1D). In sum, dSETD3 does not appear to regulate mTORC1 activity or cell proliferation in S2 cells.

### Subcellular localization of dSETD3

**Fig 2:**
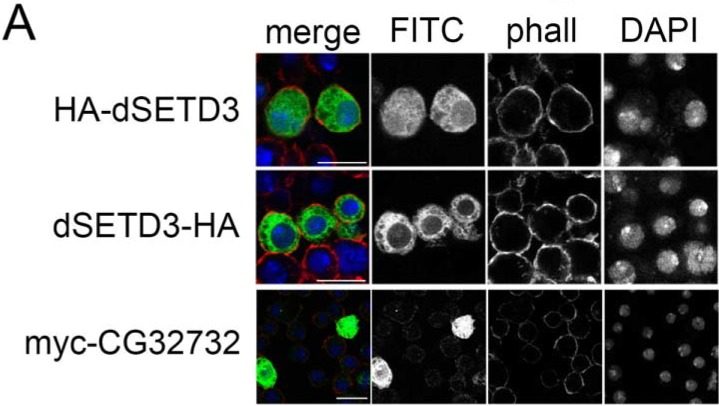
dSETD3 is localized in the nucleus and the cytoplasm. **(A)** Epitope tagged dSETD3 is present in the cytoplasm and the nucleus. S2 cells were transfected with constructs to express either N-terminally or C-terminally HA-tagged dSETD3, and then immunostained for HA-tag or myc-tag (green “FITC”), DAPI (blue) and phalloidin (red “phall”) to stain the actin skeleton. Scale bar: 10μm.

Since dSETD3 has been described to have both histone and non-histone targets, we checked the localization of overexpressed HA-tagged dSETD3 in S2 cells. Interestingly, the majority of SETD3 protein localized to the cytoplasm, with some SETD3 protein also visible in the nucleus (Fig 2A). We observed this localization with either N-terminally or C-terminally tagged dSETD3 (Fig 2A), for both HA and myc-tagged protein (Fig 2A).

### Generation of dSETD3 knockout flies

**Fig 3:**
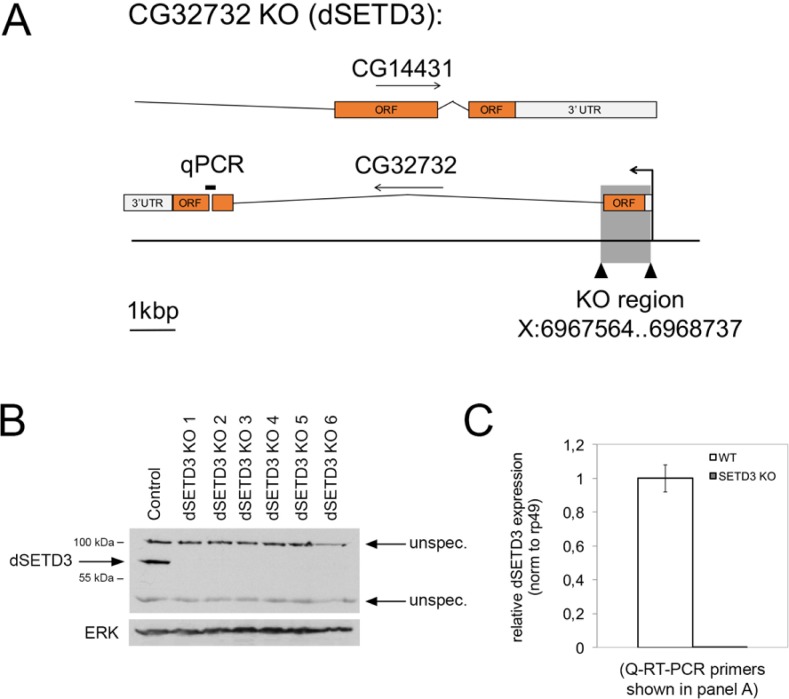
Generation of dSETD3 KO flies. **(A)** Schematic of the dSETD3 genomic locus and the knockout region. Orange indicates coding exons. To avoid interfering with the splicing of CG14431 only the first exon of CG32732 (dSETD3) was removed and replaced with a dsRED expression cassette using CRISPR-induced homologous recombination. The location of the amplicon used for Q-RT-PCR in panel C is shown and labeled with ‘qPCR’. **(B)** Complete loss of dSETD3 protein in different dSETD3 KO lines. Knock-out stock 2 was used in this study. Homozygous flies were lysed and analyzed by immunoblotting with purified dSETD3 antibody. **(C)** dSETD3 mRNA is completely lost in dSETD3 KO flies. mRNA was isolated from control and dSETD3 KO homozygous flies and dSETD3 expression was analyzed by RT-qPCR against the region indicated in (A), and normalized to rp49. Error bars: standard deviation (SD).

To better study the *in vivo* function of dSETD3, we generated dSETD3 knockout (KO) flies (Fig 3A). Of note, there is another gene, CG14431, that is encoded on the opposite strand of dSETD3 and partially overlaps with dSETD3. Using CRISPR-Cas9 we induced two double-stranded breaks in the genome, one directly downstream of the transcription start site of dSETD3 and the second at the end of the first exon of dSETD3 (CG32732) (Fig 3A). This removes the translation start site of dSETD3 as well as circa half of the coding sequence, leading to a predicted null allele, without removing any coding region of CG14431. Using homologous recombination, we replaced this region with a dsRED expression cassette. We screened flies for dsRed expression and confirmed the dSETD3 knock-out by immunoblotting for dSETD3 (Fig 3B) and by Q-RT-PCR using a downstream primer pair (location shown in Fig 3A) on RNA isolated from homozygous KO flies (Fig 3C). CG14431 expression is not reduced in dSETD3 knock-out animals (S3A Fig).

### dSETD3 knockout flies are viable and have no strong phenotypes

**Fig 4:**
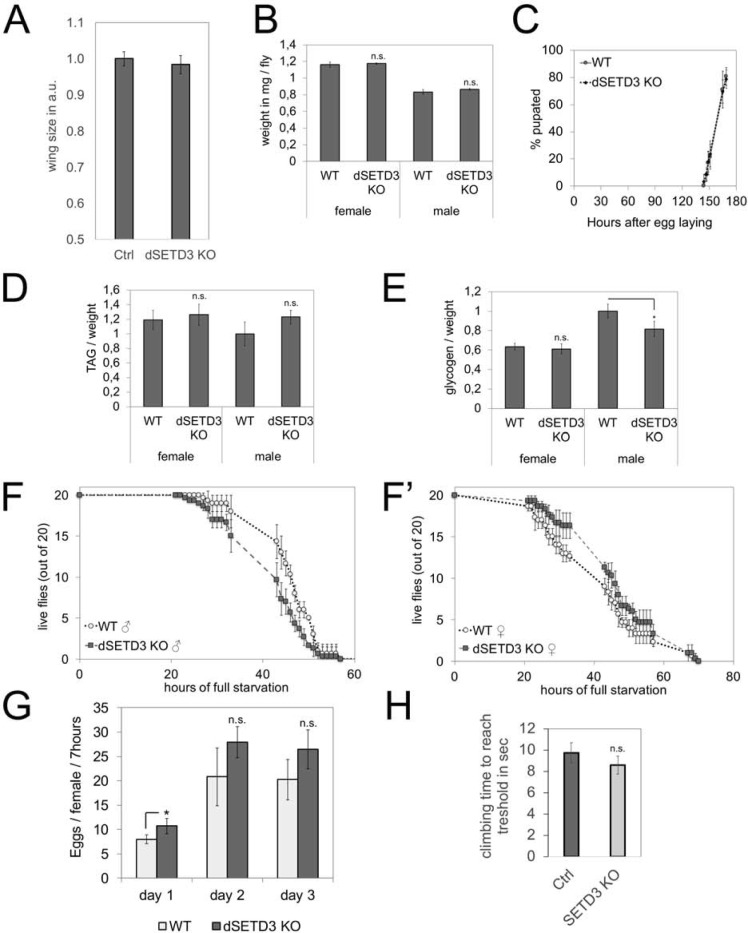
Phenotypic characterization of dSETD3 KO flies. **(A)** Wing size of adult female control (n = 7) and dSETD3 KO (n = 8) flies. Error bars: standard deviation (SD). **(B)** Weight of control and dSETD3 KO flies (n = 4 x 8 flies for each condition). Error bars: standard deviation (SD). **(C)** Pupation timing of control and dSETD3 KO animals at 25°C (n = 8 x 30 flies for each condition). Error bars: standard deviation (SD). **(D)** Fat storage measured by TAG abundance in control and dSETD3 KO flies, normalized to total body weight (n = 4 x 8 flies for each condition). Error bars: standard deviation (SD). **(E)** Glycogen storage measured in control and dSETD3 KO flies, normalized to total body weight (n = 4 x 8 flies for each condition). Error bars: standard deviation (SD); * *t* test < 0.05. **(F-F’)** Full starvation of control and dSETD3 KO animals on PBS/agarose (0.7%) for male (F) or female (F’) adult flies (n = 3 x 20 flies for each condition). Error bars: standard deviation (SD). **(G)** Fertility of control and dSETD3 KO mated females over the course of three days (n = 4 x 8–10 flies). Error bars: standard deviation (SD); * *t* test < 0.05. (H) dSETD3 KO flies do not have impaired motility, assayed using a climbing assay with control and dSETD3 KO adult females. Flies were put into plastic tubes, tapped down, and observed climbing towards a light source at the top of the tube. The time was measured that was required for 50% of the flies in one tube to pass a set threshold (biological quadruplicates, each measured twice). Error bars: standard deviation (SD).

dSETD3 knockout animals are homozygous viable with no obvious patterning defects that we could find. To test if dSETD3 knockouts have more subtle defects, we assayed growth and metabolic phenotypes. dSETD3 KO flies showed no difference in wing size or body weight compared to control flies (Fig 4A and 4B). Furthermore, their developmental rate, assayed as the timing from egg laying to pupation when animal growth is completed, was also normal (Fig 4C). Together, these data indicate that the growth rate of dSETD3 KO animals is normal. This was surprising to us because knockdown of SETD3 using two different RNAi lines caused a reduction in tissue size (Fig 1A and 1B). Thus, we repeated the knockdown of dSETD3 in the posterior part of the wing in SETD3-KO flies to test whether the knockdown phenotype is due to off-target effects. dSETD3 knockdown caused a similar reduction in wing size and cell size in the dSETD3 knock-out background (S3B and S3C Fig) as it did in the control background (Fig 1A and 1B), suggesting that both independent non-overlapping RNAi lines cause tissue undergrowth due to off-targets effects. One of the RNAi lines overlaps with the 3’UTR of CG14431 whereas the other one does not, ruling out CG14431 as a common off-target of both lines. We conclude that the observed tissue and cell size effects upon dSETD3 knockdown are not specific to dSETD3 but more likely should be attributed to off-target effects.

dSETD3 KO flies also showed no difference in fat storage (Fig 4D). Whereas virgin female dSETD3 knockouts had normal glycogen levels, dSETD3 KO males consistently had slightly lower glycogen levels than controls (Fig 4E). As a sensitive measure for the ability of the flies to mobilize their nutrient stores, we challenged animals with complete food deprivation. Animals that have reduced nutrient stores or impaired nutrient mobilization capacity display reduced survival curves in response to complete starvation [39, 40]. Both male and female dSETD3 knockout animals, however, had normal survival in response to full food deprivation (Fig 4F and 4F’). (We repeated this experiment 3 times with no consistent changes in survival between knockout animals and controls.) In addition, mated dSETD3 KO females also did not show a consistently significant change in fertility (Fig 4G).

### Transcriptional changes in dSETD3 knockouts

**Fig 5:**
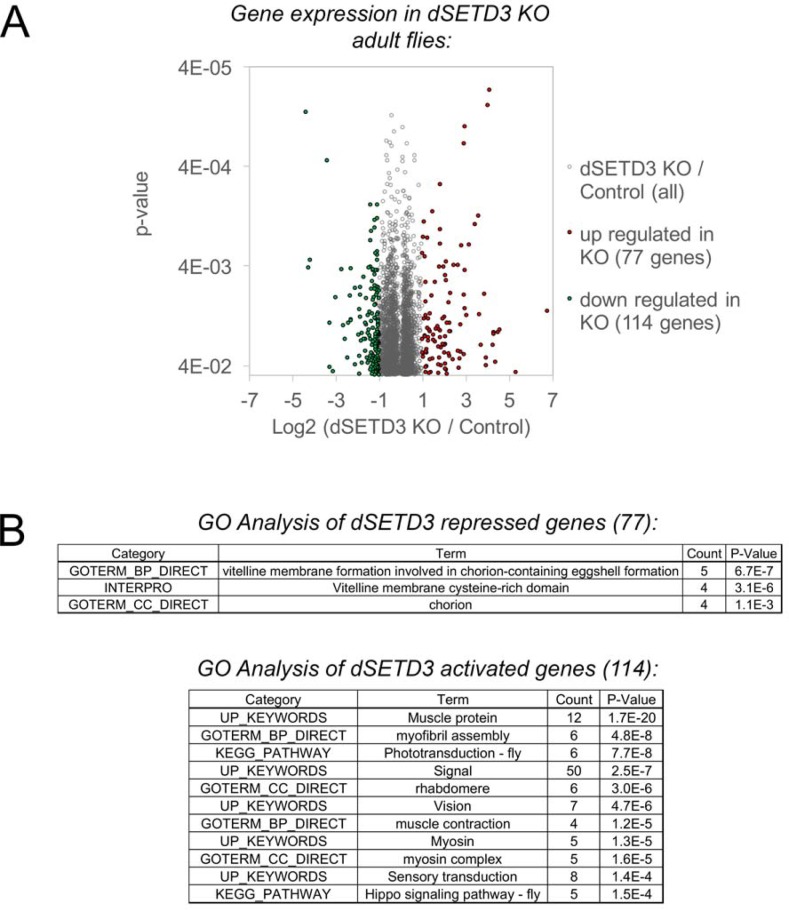
Transcriptional effects of dSETD3 KO in adult virgin flies. **(A)** Control and dSETD3 KO virgin female flies were lysed in TRIZOL, RNA extracted and subjected to microarray analysis. Volcano blot shows all genes that were statistically significantly up or downregulated (*t* test < 0.05). Genes that were up or downregulated at least 2-fold upon dSETD3 KO (*t* test < 0.05) are indicated in green (down) and red (up). Data are available under GEO accession number GSE113846. **(B)** Gene ontology analysis using DAVID of dSETD3-affected genes [41, 42].

Since dSETD3 is partially localized in the nucleus and hSETD3 has been shown to interact with several transcription factors, we assessed global changes in mRNA levels in dSETD3 knockout virgin female flies compared to controls via microarray analysis (Fig 5A and S1 Table). We found 77 genes whose mRNA levels were upregulated in the dSETD3 KO flies and 114 genes whose expression was downregulated in dSETD3 KO flies (Fig 5A and S2 Table). Gene ontology (GO) enrichment analysis on these genes revealed that the upregulated genes were slightly enriched for genes that are involved in vitelline membrane formation. Genes downregulated in the dSETD3 KO were enriched for genes annotated as muscle proteins (Fig 5B). This is interesting as SETD3 was shown to play a role during muscle differentiation in mouse C2C12 cells [25]. To test whether dSETD3 KO mutant might have impaired muscle function we performed a climbing assay. We did not observe a significant difference in climbing ability between control and dSETD3 KO adult flies (Fig 4H).

### Hypoxia response of dSETD3 knockout animals

**Fig 6:**
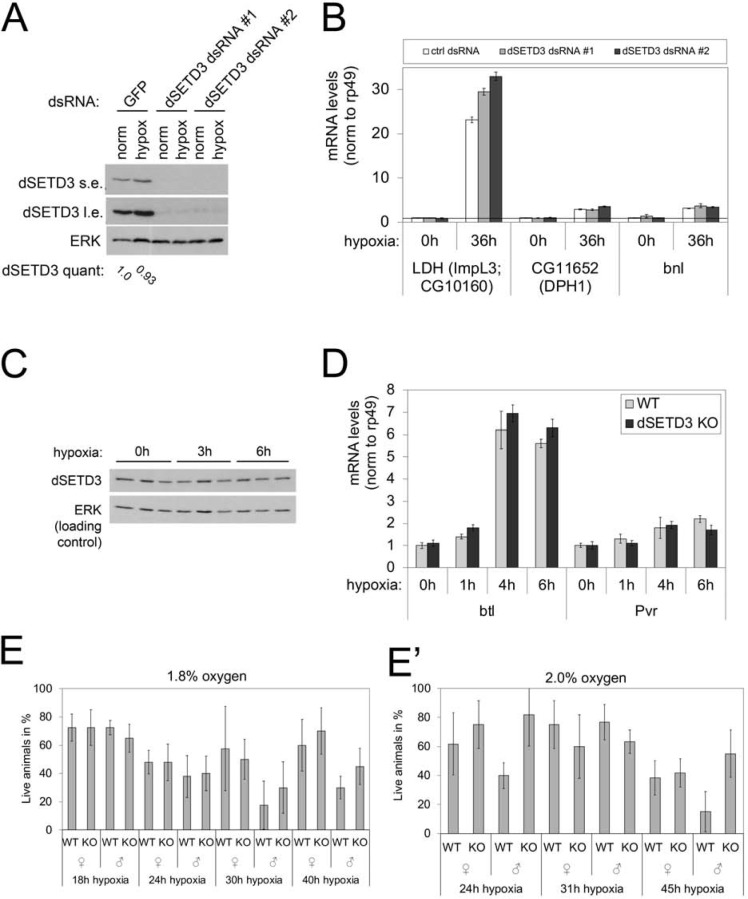
dSETD3 does not play a role in hypoxia response in *Drosophila*. **(A)** dSETD3 protein levels do not change upon hypoxia in S2 cells. S2 cells were treated for 5 days with control or dSETD3 dsRNA and then reseeded and subjected to control or hypoxic conditions (1% oxygen) for 36 hours. Cell lysates were analyzed by immunoblotting with indicated antibodies. Quantification of dSETD3 bands normalized to ERK bands was done with ImageJ [46]. **(B)** Expression of hypoxia induced genes in S2 cells is not dependent on dSETD3. S2 cells were treated for 5 days with control or dSETD3 dsRNA and then reseeded and subjected to control or hypoxic conditions (1% oxygen) for 36 hours. RNA was isolated and analyzed by RT-qPCR. Error bars from technical triplicates: standard deviation (SD). **(C)** dSETD3 protein levels do not change upon hypoxia in flies. Control flies were subjected to hypoxia (5% oxygen) for 3 or 6 hours and lysates were analyzed by immunoblotting with indicated antibodies. Three samples (5 larvae each) per condition are shown. **(D)** Expression of hypoxia induced genes is not affected by dSETD3 KO in flies. Control and dSETD3 KO male flies were subjected to 1, 4 or 6 hours of hypoxia (1.8% oxygen). RNA was isolated and analyzed by RT-qPCR. Error bars from technical triplicates: standard deviation (SD). **(E-E’)** dSETD3 is not required for survival during hypoxic conditions. Control and dSETD3 KO flies were put into hypoxic conditions (1.8% oxygen (E, n = 4 x 10 flies) and 2% oxygen (E’, n = 6 x 10 flies)) for indicated amounts of times and surviving animals after the treatment were counted. Error bars: standard deviation (SD).

Since SETD3 protein levels decrease in response to hypoxia in human HeLa cells and SETD3 regulates VEGF expression under hypoxic conditions [27], we aimed to test whether dSETD3 plays a role in the response of S2 cells or adult flies to hypoxia. We first asked whether dSETD3 affects the ability of S2 cells to mount a transcriptional response to hypoxia. To this end, we knocked-down dSETD3 expression in S2 cells using dsRNA and induced a hypoxia response by incubating cells for 36 hours in normoxic or hypoxic conditions (1% oxygen). Knockdown of dSETD3 was highly efficient, assayed by immunoblotting (Fig 6A). We tested expression of three genes that are known to be upregulated by hypoxia in S2 cells, Lactate dehydrogenase LDH (ImpL3) [43], Dph1 (CG11652) [43] and branchless (bnl) [44]. All three genes were transcriptionally induced upon hypoxia, and control (GFP) and dSETD3 knockdown cells showed a similar induction of all three genes (Fig 6B). We also did not detect any changes in dSETD3 protein levels in S2 cells in response to hypoxia (Fig 6A). Next, we analyzed dSETD3 protein levels in flies and found that hypoxia (5% oxygen) does not affect dSETD3 protein levels (Fig 6C). Similar to what we found in S2 cells, dSETD3 knock-out flies induced hypoxia targets genes btl [45] and Pvr to a similar extent as control flies (Fig 6D). We also tested the survival of dSETD3 KO flies under hypoxic conditions. Both at 1.8% and 2% oxygen, control and dSETD3 KO flies survived hypoxia to a comparable degree as control animals (Fig 6E and 6E’). In sum, we could not find any obvious phenotypes in dSETD3 knockout animals or cells in response to hypoxia.

## Discussion

We have generated and characterized a complete knock-out model of dSETD3 in Drosophila. To our knowledge this is the first time that this protein has been studied at an organismal level and the first time a complete loss of function of SETD3 in an animal has been generated.

Although we anticipated phenotypes related to cell growth and proliferation based on earlier reports from cell culture, we unfortunately did not find any growth phenotypes in dSETD3 KO flies. This was also surprising given that we saw reduced tissue growth in the wing when knocking down dSETD3 using two independent dsRNA constructs. Given that the two dsRNAs are non-overlapping, this is usually taken as a strong indication that the phenotype is on-target. Hence the knockdown and the knockout data were difficult to reconcile. By performing the knockdowns in a dSETD3 knock-out background we still observed the same growth phenotypes, which leads us to believe that the two RNAi lines used in our study are producing growth phenotypes due to off-target effects.

We also hypothesized that dSETD3 KO would show phenotypes related to hypoxia, because human SETD3 was recently linked to hypoxia-response in HeLa cells [27]. Unfortunately, however, we also could not find any hypoxia-related dSETD3 knockout phenotypes in *Drosophila*. This raises the question why we did not find phenotypes in Drosophila related to the ones described in human cell culture. One possibility is that the function of SETD3 is not conserved between flies and mammals. Another explanation might be that the core, evolutionarily conserved function of SETD3 is not related to hypoxia or to growth, but rather to another biological function that still remains to be uncovered. For this reason, we wanted to publish the current report, so that the community knows about the availability of these knockout flies, so they can be obtained from us to study other phenotypes of interest. A third option is that SETD3 function may be genetically redundant with the function of another methyltransferase in the fly and perhaps also in other organisms. That said, SETD3 has been evolutionarily maintained between flies and humans, therefore it must have some non-redundant function in the animal. This function, however, might be quantitative rather than qualitative. Hints in this direction might lie in the gene expression changes we presented here, where indeed circa 200 genes change in expression in the knockout flies. Future work will hopefully shed additional light on these issues.

## Materials and methods

### Fly stocks and maintenance

Experiments and maintenance of flies were performed under a 12hr/12hr light/dark cycle at 25°C at constant humidity on cornmeal food [47]. As control flies we used isogenic w[1118] flies from Bloomington. The dSETD3 KO allele was backcrossed to w[1118] at least five times. Flies that were used for growth measurement and metabolic assays were all reared on standard controlled density before the experiment. Following chromosomes and stocks were used: hhGAL4 (II); UAS-GFP(III), eng-Gal4 (II), dSETD3 RNAi 1 is a KK-line against CG32732 from VDRC (104185), dSETD3 RNAi 2 is a GD-line against CG32732 from VDRC (51749).

### Fly food

Flies were grown and maintained on food consisting of the following ingredients for 30 liters of food: 240g agar, 660g sugar syrup, 2400g malt, 2400g corn meal, 300g soymeal, 540g yeast, 72g nipagin, 187mL propionic acid and 18.7 mL phosphoric acid.

### Metabolic measurements

TAG and glycogen measurements were essentially as in [40]. We seed 60 1st instar larvae per vial to grow under defined density conditions. Hatching adults were collected within a 12-hour window, and allowed to age 3 or 4 days as indicated. Three times 8 animals were crushed in 500μl 1xPBS with 0.05% Tween-20 (Applichem, A1389) and heat-inactivated for 5 minutes on 70°C. 100μl lysate were cleared of debris and used for Protein concentration measurement using Bradford reagent (BIORAD protein assay, 500–0006). 200μl lysate were used for TAG measurement by adding 2μl of Lipase (Calbiochem, cat 437707) to the lysate, incubating at 37°C ON, spinning down debris and analyzing by using the free glycerol reagent from Sigma (F6428). For glycogen measurement, another 200μl of the original lysate were split into two times 30μl–a control lysate and one lysate to which 1 μl of amyloglucosidase solution (14 U per μl, Sigma 10115) was added. After 1h incubation at 50°C, both lysates were analyzed using the Glucose reagent from Sigma (G3293) and subtracting the glucose content of the control lysate from the amyglucosidase lysate to yield relative glycogen levels of each sample.

### Starvation treatments

For starvation survival, adult 5-day old flies were put on 0.7% Agarose diluted in 1x PBS. We put 20 flies in one normal fly food vial and used biological triplicates for each measurement and condition. Flies were regularly counted until all flies have died.

### CRISPR dSETD3 KO generation

Homology arms 1 and 2 were PCR amplified using the oligos mentioned below in the ‘Oligo’ section, and then cloned into pHD-pDsRed-attP (addgene #51019) via AarI (arm1) and SpeI-XhoI (arm2) restriction sites. Two guide RNAs were each cloned into pU6-BbsI-chiRNA (addgene #45946) via BbsI. The donor vector and both gRNA vectors were injected together into nos-Cas9 embryos (Bloomington 54591). Hatching flies were crossed to balancer stocks and resulting F1 flies were screened for dsRED fluorescence.

### Wing size and cell size measurement

50 L1 larvae were collected from an apple plate into one vial. Hatching adults were kept at 25°C for 3 days before flies were put into preservation medium (70% EtOH and 30% glycerol). Wings were then mounted in Hoyer’s medium and imaged. The size of the wing’s posterior and anterior compartments (green area and non-shaded area in S2 Fig, respectively) were analyzed with ImageJ [46]. To measure cell size, a region of fixed area (encompassing roughly 100 trichomes) was placed in the wing between veins 4 and 5, and the number of trichomes within this area were counted. Cell size was calculated as the total area of the region divided by the number of cells in this region.

### Fertility assay

10 wild type control or dSETD3 KO female virgins were mated with 7 males and put together with the males into an embryo collection cage. Females were allowed to lay eggs for 7 hours, several days in a row. Eggs were then manually counted for each day and normalized to number of females in the cage.

### Motility assay

Control and SETD3 knock-out flies were grown in a density controlled manner and adults were aged for 5 days. 20 female flies were put into individual tubes. Flies from one tube were then transferred into a plastic tube without anesthetizing. The plastic tube was tapped to collect all flies at the bottom of the tube. Flies were then videotaped climbing up the plastic tube towards a light source on top of the tubing. The time 50% of the moving flies needed to reach a certain threshold was measured twice for the same flies. Each genotype was measured in 4 biological replicates. Control and dSETD3 knock-out flies were handled in parallel.

### Cell culture

*Drosophila* S2 cells were cultured at 25°C in Schneider’s *Drosophila* Medium (GIBCO 21720), supplemented with Penicillin/Streptomycin and 10% FCS. dsRNA was generated by performing a T7 transcription reaction from an amplified genomic region of the respective gene. Gene knockdowns were done by treating S2 cells with 12μg/ml dsRNA in serum-free medium for one hour. Cells were then given serum-containing medium and allowed to grow for 5 days before analysis, to allow for the knockdown to take effect.

### Hypoxia experiments

All Hypoxia experiments were performed in an hypoxia incubator from NUAIRE (NU-5841) at 25°C for various amounts of time as indicated in the figure legends.

### Immunostainings

S2 cells or wing discs were fixed using 4% formaldehyde in 1xPBS for 20 min, blocked with 0.1% BSA and 0.2% TritonX-100 in 1xPBS, stained with indicated antibodies overnight. After staining with secondary antibodies cells or tissues were mounted in a glycerol-based mounting medium. Images were recorded using a Leica SP8 confocal system with a 63x or 40x objective.

### RNA and quantitative RT-PCR

RNA was isolated using TRIZOL reagent (Ambion, 15596018). Reverse transcription was done with RevertAid Premium Reverse Transcriptase (Thermo Scientific, EP0732). Q-PCR was done with Maxima SYBR Green/ROX master mix (Thermo Scientific, K0223). Genes were normalized to rp49 levels.

### Antibodies

Following Antibodies were used in this study: guinea-pig anti-CG32732 (dSETD3) was self-made in out laboratory by immunization of guinea-pigs with full length 6xHis-CG32732. Rat anti-HA tag (clone 3F10, Roche, 11 867 423 001), rabbit anti-pS6K(T398) (Cell Signaling, 9209), self-made guinea pig anti-S6K [47], rabbit anti-ERK1/2 (Cell Signaling, 4695).

### Data and analyses

Microarray data are deposited at NCBI Geo with Accession GSE113846.

Statistical significance in the figures was calculated using Student’s t tests.

### Oligos

Sequences for the oligos used in this work are provided in Table 1.

**Table 1:** Oligo sequences.

qPCR—mRNA expression levels	
GCTGTTAACACTGGAGAACAG	CG32732 –fw
TCTACGAAACCATTGTGCAC	CG32732 –rev
ACCTCCGTTTTGGGCGA	LDH (ImpL3; CG10160)–fw
ATGTTGGCGGACTTCTGC	LDH (ImpL3; CG10160)–rev
CCCGTTGCTAAAGGCTTA	CG11652 –fw
CGTCCAGGCGCAGATTCT	CG11652 –rev
CAAGACGGTGCATTCATCG	btl–fw
CTTGGATATCCCTCGCCAG	btl–rev
GTGAATTCACAATTCTCCAGC	bnl–fw
CAGAGATACAGGCAAGTGG	bnl–rev
TAGTGATATCAGATCTGCCCG	Pvr–fw
ACAGACCGATCAGGATGAG	Pvr–rev
CAATAATTGCAAGCGAAGCA	CG14431—fw
TGCAAGTGTTTTTGCTCCTG	CG14431—rev
dsRNA	
gccattaatacgactcactatagggGCAGCCACAACAAAACGGAGT	dSETD3 #1 –fw
gccattaatacgactcactatagggGGCTATCTCCAGTCCCTCACT	dSETD3 #1 –rev
gccattaatacgactcactatagggAGGGCAAGCATTCTGGACAAG	dSETD3 #2 –fw
gccattaatacgactcactatagggGTGGGTTGTCTGCTTAAATCC	dSETD3 #2 –rev
gccattaatacgactcactatagggCTGCCTGCCAAATACAACACC	dSETD3 #3 –fw
gccattaatacgactcactatagggCCCGTCCTCTGATTCTTGTTT	dSETD3 #3 –rev
dSETD3 KO generation	
gccaCACCTGCaactTCGCAGCACGAGATAAAGACGAAA	homology arm 1 –fw
gccaCACCTGCaactCTACAGTTGGCCTAGAAATAGAAAT	homology arm 1 –rev
gccaactagtGTGTGGCCATTCGTCGG	homology arm 2 –fw
gccactcgagAGGATGCAGTGTATAAGCTATT	homology arm 2—rev
cttcGCTCTGAAGATCTTTTCAGT	gRNA for homology arm 1—fw
aaacACTGAAAAGATCTTCAGAGC	gRNA for homology arm 1—rev
cttcgCGACGAATGGCCACACTGC	gRNA for homology arm 2—fw
aaacGCAGTGTGGCCATTCGTCGc	gRNA for homology arm 2
GGGTGCTTCACGTACACCTT	Test for dsRED presence—fw
TGATGTGCCAAAAATGCAAC	Test for dsRED presence

Oligos used in this study.

## Supporting information

S1 Fig CG32732 is the Drosophila ortholog of human SETD3. **(A)** Protein sequence of *Drosophila* dSETD3 (NP_727144.1) and human SETD3 (NP_115609.2), aligned using Clustal Omega software [36, 48].
**(B)** Several human SET-domain containing proteins were aligned with CG32732 and then submitted to phylogenic tree analysis in Clustal Omega [36] and visualized with Dendroscope Software [49].
(PSD)

S2 Fig EnGAL4 and hhGAL4 expression domains. Representative image of a wildtype *Drosophila* wing, with the posterior compartment colored green to indicate the enGAL4 and hhGAL4 expression domain.
(PSD)

S3 Fig CG14431 expression and dSETD3 knockdown in dSETD3 KO flies. (A) mRNA levels of CG14431 in control and dSETD3 KO flies, quantified by Q-RT-PCR, normalized to rp49. Error bars: standard deviation (SD). *t-test = 0.045.
(B-C) Knockdown of dSETD3 in dSETD3 KO flies in the posterior compartment of the wing decreases tissue size, indicating it is an off-target effect. (B) Size of the posterior compartment normalized to size of the control anterior compartment. (C) Cell size in the posterior compartment was determined by counting the number of cells via their trichomes in a defined area, and then calculating the inverse–ie area per cell. Animals were raised at 25˚C, 10 wings per genotype were used for quantifications. Error bars: standard deviation (SD); *** *t* test < 0.001
(PSD)

S1 Table Expression values of all *Drosophila* genes in control and dSETD3 KO female flies. (XLSX)

S2 Table Differentially expressed in genes in dSETD3 KO female flies. This table contains all microarray probes whose expression was at least 2 fold different between control and dSETD3 KO flies with a p-value smaller than 0.05.
(XLSX)
